# High-throughput sequencing of microbial community diversity in soil, grapes, leaves, grape juice and wine of grapevine from China

**DOI:** 10.1371/journal.pone.0193097

**Published:** 2018-03-22

**Authors:** Yu-jie Wei, Yun Wu, Yin-zhuo Yan, Wan Zou, Jie Xue, Wen-rui Ma, Wei Wang, Ge Tian, Li-ye Wang

**Affiliations:** 1 College of Food Science and Pharmacy, Xinjiang Agricultural University, Urumqi, China; 2 China National Research Institute of Food & Fermentation Industries, Beijing, China; University of Torino, ITALY

## Abstract

In this study Illumina MiSeq was performed to investigate microbial diversity in soil, leaves, grape, grape juice and wine. A total of 1,043,102 fungal Internal Transcribed Spacer (ITS) reads and 2,422,188 high quality bacterial 16S rDNA sequences were used for taxonomic classification, revealed five fungal and eight bacterial phyla. At the genus level, the dominant fungi were *Ascomycota*, *Sordariales*, *Tetracladium* and *Geomyces* in soil, *Aureobasidium* and *Pleosporaceae* in grapes leaves, *Aureobasidium* in grape and grape juice. The dominant bacteria were *Kaistobacter*, *Arthrobacter*, *Skermanella* and *Sphingomonas* in soil, *Pseudomonas*, *Acinetobacter* and *Kaistobacter* in grape and grapes leaves, and *Oenococcus* in grape juice and wine. Principal coordinate analysis showed structural separation between the composition of fungi and bacteria in all samples. This is the first study to understand microbiome population in soil, grape, grapes leaves, grape juice and wine in Xinjiang through High-throughput Sequencing and identify microorganisms like *Saccharomyces cerevisiae* and *Oenococcus* spp. that may contribute to the quality and flavor of wine.

## Introduction

Microbial biodiversity is highly essential for harboring a healthy environment with sustainable economy, especially at agricultural field. In recent years, several studies on biodiversity tried to characterize the microbiome in different agricultural ecosystems, as to understand the dynamics of plant and microbe interaction. It is discovered that plant-associated bacteria and fungi colonize on both exterior (epiphytes) and interior surfaces (endophytes) of plants, while the surrounding soil around the plant acts as the major resource for these microbes [1]. As plant species and soil type play a predominant role in soil microbial community, the interaction between plant and soil microbes are highly complex. *Vitis vinifera* (wine grape) is an economically important agricultural crop. *V*. *vinifera* phyllosphere is easy to be colonized by both bacteria and fungi, which in-turn modulates grapevine health, development, and grape qualities [2]. In this case, microbial activity plays a critical role on grape production and quality [2–3].

Due to its nutritive contents like organic acids, amino acids, and trace elements, wine does have beneficial effects on human health. Studies have shown that long term in-take of wine in diet could prevent chronic diseases and cardiovascular diseases [4–7]. The winemaking is a composite process where numerous microorganisms were involved, especially yeast and bacteria [8–11]. Understanding the composition and population dynamics of the microbial population throughout brewing is highly essential for controlling the process, thereby improving the quality and safety of the wine [12–13]. Microbes in wine brewing is mainly from wine grapes, vineyards, brewing equipment, and surrounding environment, impacts the quality of the wine at a larger level [14–15]. In recent years, studies have shown that the unique flavor in wine is mainly generated from the microbial metabolic process during wine making process [16–17]. Therefore, selecting specific microbes for wine fermentation can help to crease the flavor, thereby enhancing the taste and quality of wine.

Current methods for microbial diversity analysis mainly include traditional culture method and non-culture methods. Traditional culture methods such as restriction fragment length polymorphism (RFLP) and random amplified polymorphic analysis (RAPD) are based on polymerase chain reaction (PCR) techniques. Compare to non-culture methods, they are laborious and time consuming, also poor in reliability. Non-culture methods mainly involve denaturing gradient gel electrophoresis (DGGE), real-time fluorescence PCR, and fluorescence in situ hybridization (FISH). They have the capability to detect the dynamic changes of microorganisms in the fermentation process, but they can only detect a certain group of microorganisms, thus in-turn restricts the complete understanding of the microbial community [18–20]. Compare to these methods, metagenomics approach has the ability to reveal the previously hidden diversity of microorganisms. After the extraction of total genetic material from a selected habitat, subsequent sequencing and bioinformatics analysis, metagenomics approach offers a powerful lens to investigate microbial communities in the specific habitat [12]. High-throughput sequencing technology has completely changed the past research model by providing large amount of data with high accuracy and low cost, and making it feasible to understand the microbial diversity at a much greater scale.

Due to the unique climate conditions and high microbial richness in natural environment, Xinjiang is widely known for wine grapes cultivation. Grapes in Xinjiang were mainly cultivated in Fukang, Manasi and Changji. These areas have gravel sandy loam, long sunshine duration, large temperature difference between day and night, small amount of precipitation and pest diseases, both are premium vintage area. Xinjiang region is very wide, so there are differences in geography and climate conditions in these three places. Fukang and Manasi areas have extremely low temperature in winter and extremely high temperature in summer. Affected by strong wind in early spring and early summer, frequent hail in late summer and sudden drop of temperature in fall, Manasi area may have different microbial diversity from Fukang area. Changji area has relatively milder climate. Besides temperature, microbes in Changji may also affected by the strong wind, sand and dust weather in spring and early summer. Because of the specific climate and soil conditions, Manasi area is one of the best grape production areas in China. However, Xinjiang is relatively un-development compare to other cities in China. Currently, we did not find reports about microbial resources and their biodiversity in the vineyard from Xinjiang region, interaction between microbial community and grape plants in Xinjiang region was also poorly understood.

In this study, Illumina MiSeq was used to study microbial community diversity of soil, grape, grapes leaves, grape juice and wine. The results will enhance our understanding of the microbiome in the vineyard from in Xinjiang region, and help to establish correlation between wine quality and regional microbiome.

## Materials and methods

### Sampling

Samples were collected from three different winery regions in Xinjiang province, China, named Fukang (A), Manasi (B) and Changji (C) area, both are cabernet sauvignon samples. A total of 20 samples were taken up in July and October, 2015, categorized as vineyard soils (T), grape (P), grape leaves (Y), grape juice (Z) and wine (J) (Table 1). Soil samples were collected in randomly-chosen plots around each vine root, collected at 5-10cm depth using a shovel and sieved to remove plant residues, macrofaunal, and stones. Undamaged grape samples were collected from several bunches with their pedicels attached. Grape leaves were collected from the vines to prevent cross contamination. Grape juices were sampled during grape crushing period. Wine samples from winery were collected from the final wine product. After collection in triplicates, each sample was stored immediately at -20°C for further study. The samples were collected from the private properties, permissions from the owners were received before sample collection.

**Table 1:** Sample list.

Sample types	Region and name	Time
Soil	A(T1, T3), B(T6), C(T8)	July
	A(T4, T5), B(T7), C(T9)	October
Grape leaves	A(Y1, Y5), B(Y11), C(Y15)	July
	A(Y9, Y10), B(Y13), C(Y17)	October
Grape	A(P1), B(P2)	October
Grape juice	A(Z1)	October
Wine	A(J1)	October

### Sample preparation for grape and grape leaves

4g of grape skin or grape leaves (no flesh and fruit stalk), was taken into 50mL sterilized centrifuge tube (L1), and 6mL of TENP buffer was added and mixed vigorously in vortex for 10min. The mixture was then centrifuged at 3000×g for 5min, supernatant was transferred to another 50mL centrifuge tube (L2). This step was repeated thrice, and a total of around 18ml supernatant was transferred to L2. L2 was then centrifuged at 9000×g for 10min. The supernatant was discarded and pellet was stored at -20°C for DNA extraction [21].

### Sample preparation for grape juice and wine

About 4mL of grape juice or wine were taken into 50mL sterilized centrifuge tube (L1), vigorously shake to mix, then vortex for 1min. The mixture was then centrifuged at 3000×g for 5min, and supernatant was discarded. For the pellet, 4mL of sterile water was added and vortexed for 1min. Mixture was centrifuged at 3000×g for 5min and supernatant was discarded. This wash step was repeater thrice, and the pellet was finally stored at -20°C for DNA extraction.

### DNA extraction and amplification

DNA from soil samples were extracted using Mag-Bind Soil DNA Kit (OMEGA) following manufacturer’s instructions. DNA from grape, grape leaves, grape juice and wine were extracted using Fast DNA SPIN Kit for Soil (MP) based on the manufacturer’s instructions. The quantity and quality of extracted DNA were assessed by spectrophotometry (Eppendorf, Germany) and agarose gel (1%) electrophoresis, respectively.

For fungal ITS regions, PCR amplification was performed using ITS 1F (5′-CTTGGTCATTTAGAGGAGTAA-3′) and ITS 1R (5′-GCTGCGTTCTTCATCGATGC-3′) as primers and genome DNA as template. PCR was performed at a final volume of 50μL mixture containing 4μL dNTPs mixture, 5μL of 10×PCR buffer (Mg^2+^ plus), 5μL of template DNA, 1μL of each primer, 0.25μL Ex Taq, and added to final volume of 50μL using ddH_2_O. PCR conditions were 3min at 98°C for initial, followed by 35 cycles at 98°C for 45s, annealing at 53°C for 30s, and extension at 72°C for 45s, and final extension at 72°C for 8 min. PCR products were stored at -20°C.

For bacterial 16s rRNA gene region, PCR amplification was performed using 16S 515F(5′-GTGCCAGCMGCCGCGGTAA-3′) and 16S 806R(5′-GGACTACHVGGGTWTCTAAT-3′) as primers and genome DNA as template. PCR was performed at a final volume of 50μL mixture containing 4μL dNTPs mixture, 5μL 10×PCR buffer (Mg^2+^ plus), 5μL template DNA, 1μL of each primer, 0.25μL Ex Taq, and added to final volume using ddH_2_O. PCR conditions were 3min at 98°C for initial, followed by at 95°C for 45s, annealing at 50°C for 30s, and extension at 72°C for 45s, and final extension at 72°C for 5 min. PCR products were stored at -20°C. Both fungal and bacterial PCR products were analyzed with 1% gel using DL2000 marker for quality examination.

### High-throughput sequencing and statistical analysis

High-throughput sequencing was performed using an Illumina Miseq platform at the International Joint Research Center of National Liquor Quality and Safety in Chinese Food Fermentation Industry Research Institute, Beijing, China. After obtaining the sequencing result and calculation of operational taxonomic units (OTUs) matrix, statistical analysis was applied using alpha indices (Shannon, Simpson, Chao 1 and ACE), heatmap of genera, principal coordinate analysis (PCoA) and UPGMA. The alpha diversity index (Shannon Index), and the species richness estimator (Chao1) were calculated by using the R package phyloseq from the OTU matrix. Principal coordinate analysis (PCoA) was performed using statistical software based on the Bray-Curtis dissimilarities calculated for the composition of the bacterial or fungal communities at the genus level.

## Results and discussion

### Abundance and diversity of members of the bacterial and fungal microbiota

Illumina Miseq sequencing generated 1,043,102 high quality fungal sequences with an average of 52,155 sequences per sample, and a total of 2,422,188 high quality bacterial sequences with an average of 121,109 sequences per sample. However, the fungal population in T1, T5, and J1 was not detected. The sequencing results were shown in Supporting Information(S1 and S2 Figs, S1 and S2 Tables). Though the rarefaction curve was not parallel with the x-axis, the Good’s coverage of fungal and bacterial reached 99.9% and 97.7% respectively, with majority of microbial diversity being captured. As we applied the Chao1 index and ACE index, it reflected the species richness of sample communities, while the Shannon and Simpson index reflected the species diversity. The Chao1 and ACE scores from 139.565 to 2941.088 and from 150.4 to 3354.01, respectively. The Shannon and Simpson scores ranged from 1.538 to 8.939, and 0.241 to 0.994, respectively.

**Fig 1:**
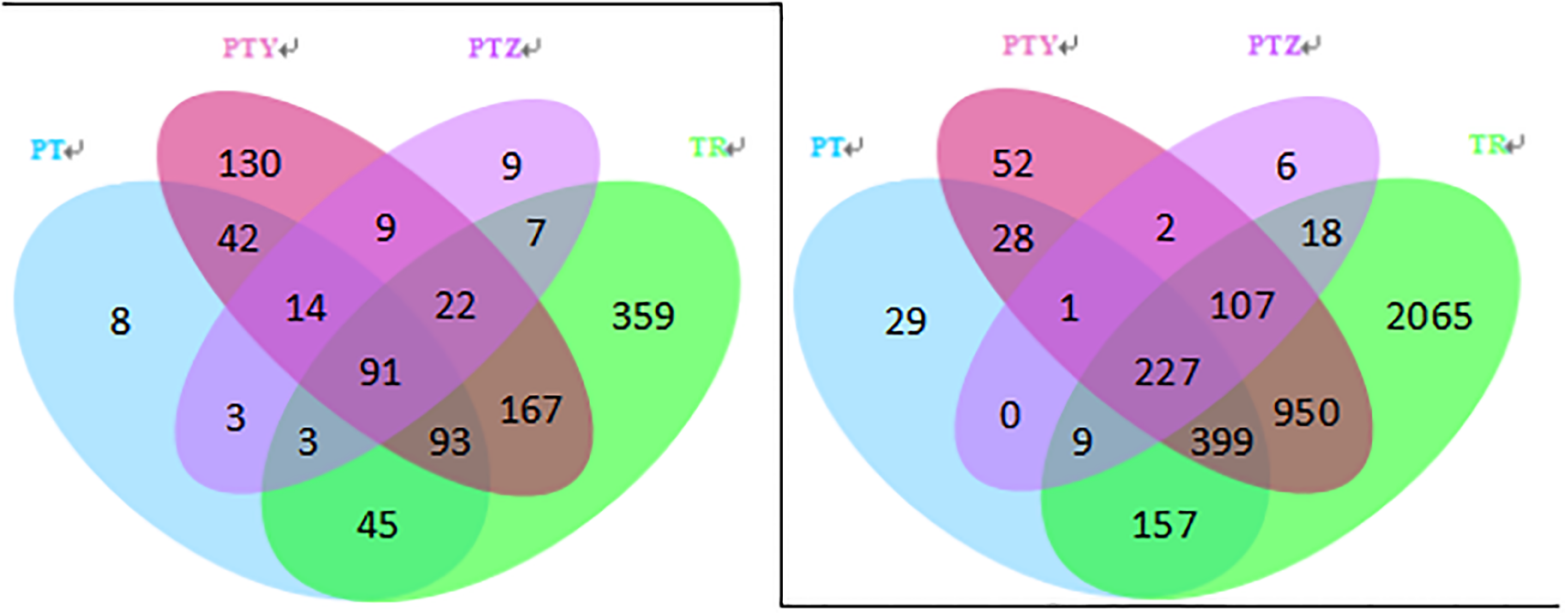
Venn of fungal (a) and bacterial (b) of T, P, Y, and Z.

Sequences were grouped into 4597 OTUs for fungi and 23,458 OTUs for bacteria (both at the 97% similarity level). As shown in Fig 1a and 1b, after removing singletons, OTUs number was 91 for fungi and 227 for bacteria, which were similar in the T, Y, P and Z samples. Fungal OTUs were 787, 568, 299 and 158. Bacterial OTUs were 3932, 1766, 850 and 370 in T, Y, P and Z respectively.

Earlier works suggested that soil provides essential nutrition for microbial growth, such as carbon sources (including amino acids, organic acids, and carbohydrates), nitrogen sources, and growth factors [22]. The physical and chemical properties of the soil, such as soil texture, mineral composition and organic matter, affect the growth and distribution of microbes [23–24]. Suitable growth environment favors the growth of most microbes, which in-turn alters the microbial community composition to larger extent in the soil and the plant [25]. And in contrast, external factors like pollution by heavy metals, organic pollutants, pesticide fertilizers, domestic sewage, and factory waste will directly alter the soil quality, causing major changes in the microbial communities [26–28]. It is described that microbes in vineyard soil are the source of primary inoculum to affect the structure of the microbial community on vine’s aerial parts [29], and this pattern is observed in our study. Diversity indices indicated that the diversity of fungal and bacterial community in T were significantly higher than Y, P, Z, J in October. The inoculation effect can be extended to wine brewing, as microbial community in P, Y and J were very similar with some minor differences according to Fig 1. Besides brewing microbes added in wine production process, these minor differences are possibly come from picking, transportation, crushing and other factors in grape crushing process, or fermenters and oak barrels in fermentation process [30–33].

The comparison of microbial communities in soil and vine plants suggests an inoculation effect between soil microbes and vine plants. But with the growth of vine plants and climate change, interaction between soil microbe and plants should be more complex. Comparison of soil samples collected from July and October indicate that fungal microbes in soil have higher diversity in July, while bacterial populations changed little in our study. As the weather turns gradually colder, temperature and humidity can inhibit fungal species that are not tolerant to these environmental challenges, while bacterial species showed higher adaptation in this environment. Besides, vine plants were found to limit the growth of bacteria by limiting nutrients in the early stage of growth [2, 5, 34–36]. And in response to biotic stress, stilbenes are produced in vine plants to effectively inhibit microbial activity [37–38]. To better understand such interaction and impact of soil microbes on vine plants, more samples should be taken gradually to provide systematic and detail results on microbial communities.

### Comparison of fungal communities in soil, grape, grapes leaves, grape juice and wine

**Fig 2:**
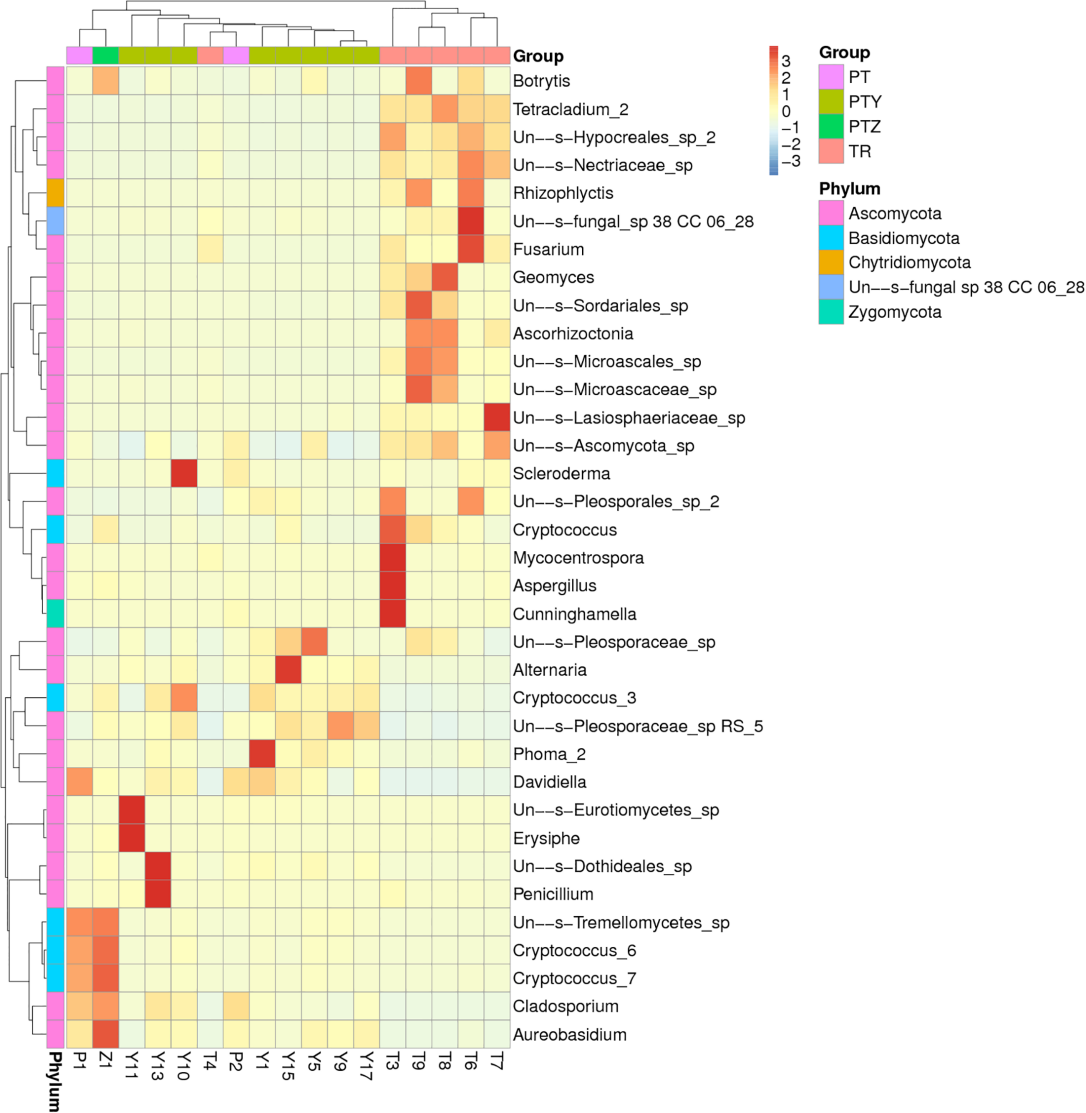
Heatmap and dendrogram of abundant fungi phyla in the microbial community of samples (excepted T1, T5 and J1).

Five fungal phyla identified were *Ascomycota*, *Basidiomycot*, *Chytridiomycota*, *Un—s-fungal sp CC 06_28* and *Zygomycota*. Heatmap revealed that *Ascomycota* and *Basidiomycot* were found as predominant phyla in T, P, Y, Z and J (Fig 2). This finding was consistent with previous reports [39–40]. Majority of OTUs in T and Y were *Ascomycota*, and similar pattern was discovered across the samples in T and Y. *Basidiomycot* remained less abundant in T and Y, but its abundance was found to be higher in P and Z samples. *Chytridiomycota*, *Un—s-fungal sp CC 06_28*, and *Zygomycota* has less abundance in T samples, but rarely detected in Y, P and Z samples. The results suggested that *Ascomycota* adapted to the T and Y environment, and *Basidiomycot* adapted to the P and Z environment.

**Fig 3:**
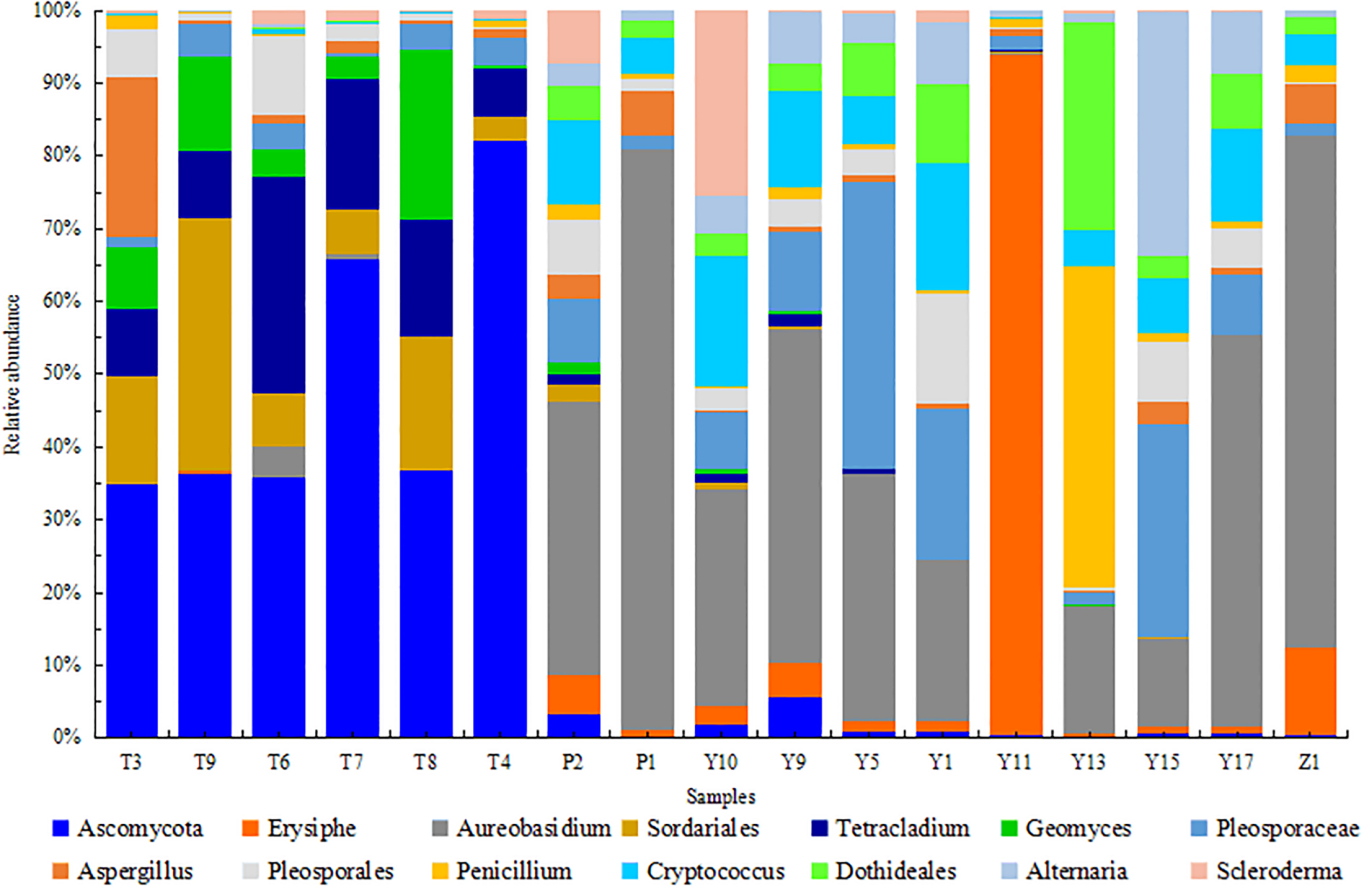
Relative abundance of fungi at genus levels of T, P, Y, and Z.

At the genus level, 271 fungal genera were detected in T, P, Y, Z and J, in which 14 fungal genera which had their relative abundance greater than 1% were selected for further analysis (Fig 3). *Ascomycota*, *Sordariales*, *Tetracladium* and *Geomyces* were the predominant genera in T samples, *Aureobasidium*, *Pleosporaceae*, *Cryptococcus* and *Dothideales* were the predominant genera in Y, P and Z samples. Other major genera were *Aspergillus*, *Pleosporales*, *Penicillium*, *Erysisphe*, *Alternaria* and *Scleroderma*. Our finding is consistent with previous reports [41]. The *Ascomycota* was sharply increased from July to October in A and B, however this was not obvious in C. In contrast, the *Sordariales*, *Tetracladium* and *Geomyces* of A, B and C were decreased in October, and *Sordariales* in C was found increased. Such difference might be due to interspecies competition. For Y samples, *Aureobasidium*, *Cryptococcus* and *Dothideales* in A, B and C have higher abundances in Octoberexcept *Dothideales* in A. In contrast, *Pleosporaceae* was found to have lower abundance in October. It is worth noticing that *Erysisphe* declined sharply in B, from 93% (Y11) in July to 1% (Y13) in October. This finding was consistent with an earlier report, which indicated that grapevine powdery mildew is one of the most damaging fungal diseases and it often occurs during July [42], suggesting that grapevine powdery mildew in B is quite serious and needs proper preventive measures [43–44]. Meanwhile, *Erysisphe* was found in Z. Interestingly, the *Penicillium* increased from 2% (Y11) to 44% (Y13), had become a dominant genus in B of Y samples in October. Consistent with other reports, *Aspergillus*, *Penicillium*, and *Alternaria* are other discovered major genera and might play an important role in T, P and J [45–46]. Studies have pointed out that ochratoxin A (OTA) produced by *Aspergillus* is a predominant global wine contaminant causing health hazards, the safe limit of OTA in wine is established at 2ug/L [47–49].

Our results indicated that fungal community changes in the grape leaves and grapes are more complex than changes in soil. This may be due to the competition between species, or natural conditions such as light intensity, light time, wind, rain, etc. or insects, human activities that causing microbial migration [29, 36]. In this metagenomics analysis, *Brettanomyces bruxellensis* has not been detected. It is able to convert hydroxycinnamic acids into volatile phenols, create ‘spicy’, ‘barnyard’, ‘animal’, ‘horse sweat’ and ‘medicinal’ odors in final wine product. Though a large number of culture-dependent techniques are available to assess the presence of this undesired yeast during the vinification processes, in several cases *Brettanomyces* is undetectable. It is been reveal that while keep alive and maintain the metabolic activities, *Brettanomyces* cells were tend to enter in a Viable But Not Culturable (VBNC) state. This could be the reason for not been detected in our wine samples.

**Fig 4:**
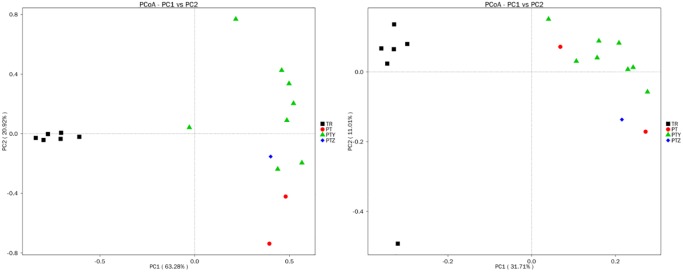
Principal coordinate analysis of fungi microbial communities of samples.

**Fig 5:**
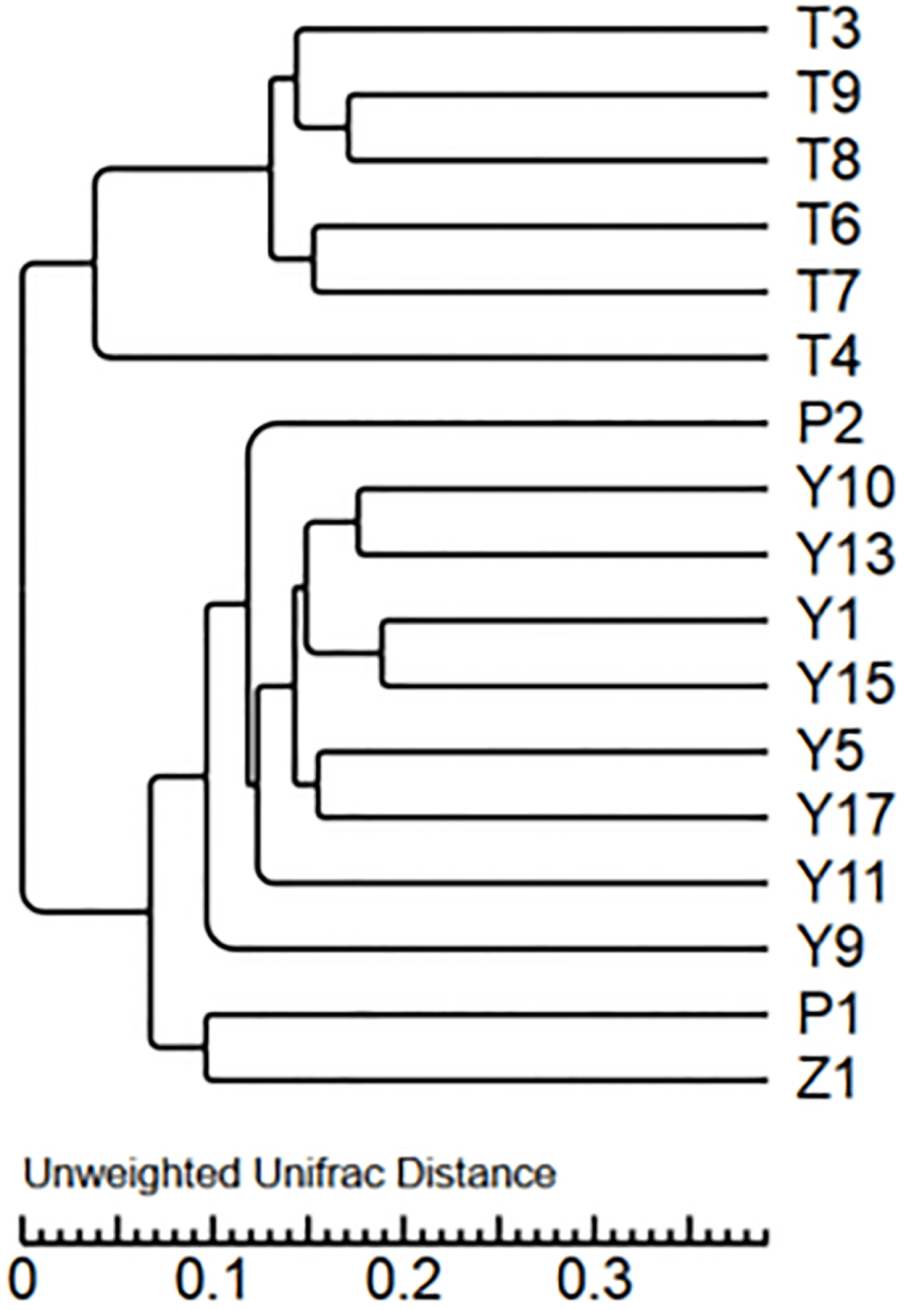
Cluster analysis of the fungal microbiota.

PCoA and cluster analyses were performed to evaluate similarities in fungal communities of T, Y, P, Z and J. One weighted (PC1 variance = 63.28%, PC2 variance = 20.92%) and another weighted (PC1 variance = 31.71%, PC2 variance = 11.01%) PCoA were performed (Fig 4). UPGMA clustering obtained a phylogenetic tree by using unweighted group averaging method (Fig 5). The branch of the sample intuitively reflected the similarity between the samples. Result indicates that same type of samples showed high similarity of fungal communities, as the soil samples and leave samples formed two big clusters. Soil samples from the same region each formed a small cluster, while similarity of leave samples from the same vineyard is relatively weaker. Compare to soil, grape leaves are highly exposed in environment. Microbes on leave are easier to be affected by natural conditions like insects, pathogens and human activities. For soil from vineyards, it is reported that fertilizing the soil, applying pesticides, no-tillage, continuous cropping and rotation, etc., affects the structure and physicochemical properties of the soil caused by human measures which in-turn change the composition and distribution of microbial communities [50–53]. The management of the vineyard may also be a factor in the composition of the microbial community.

### Comparison of bacterial communities in soil, grape, grapes leaves, grape juice and wine

**Fig 6:**
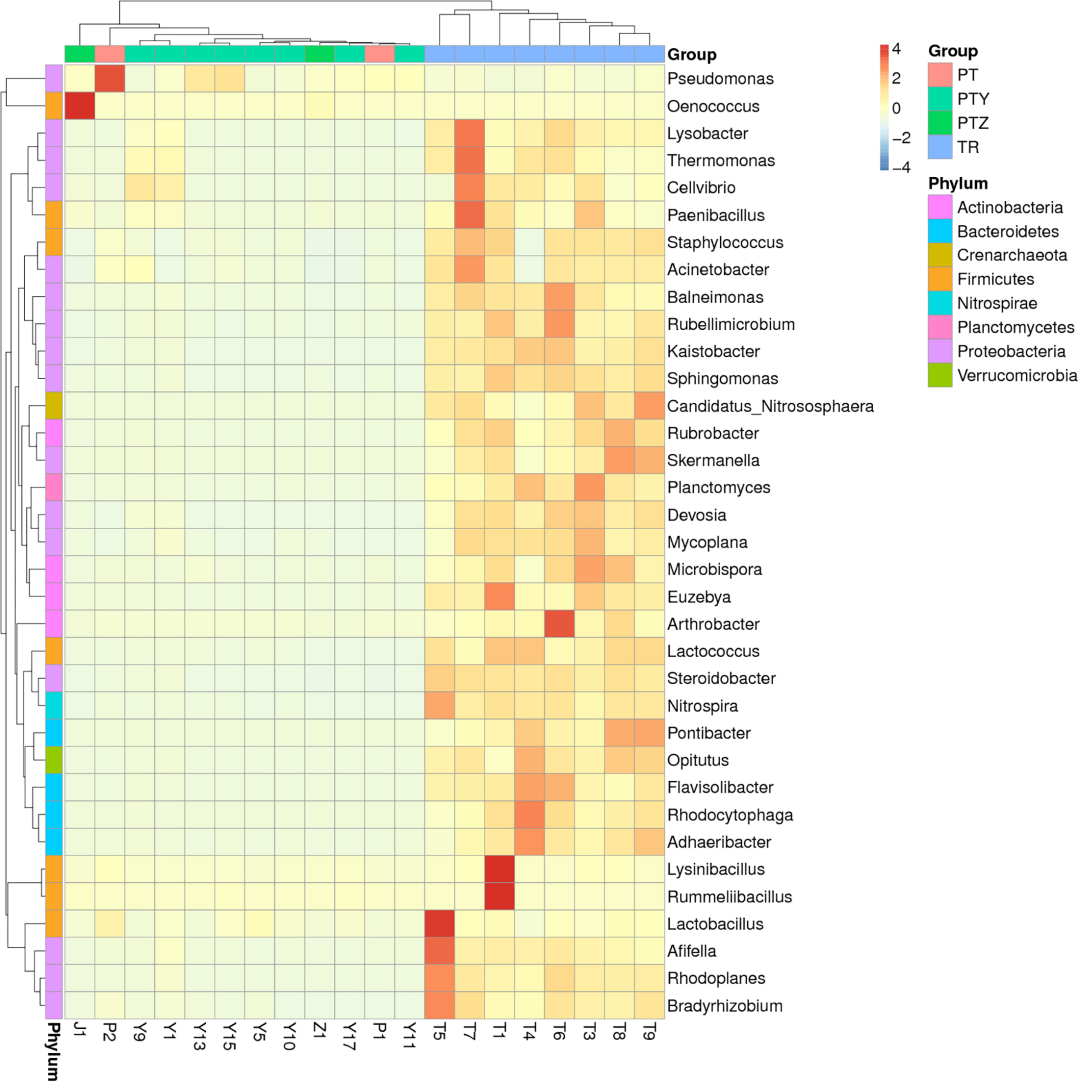
Heatmap and dendrogram of abundant bacteria phyla in the microbial community of samples.

Compared to fungal population variation, the overall diversity of the bacterial microbiota in T, P, Y, Z and J samples were higher, especially in P samples. Grapes tend to mature quickly by sunlight, rain, and other conditions, as these factors can easily cause them to rot. And in juice samples, the high sugar and nutrients can increase bacterial growth [29,36]. Detected major bacteria phyla include *Actinobacteria*, *Bacteroidetes*, *Crenarchaeota*, *Firmicutes*, *Nitrospirae*, *Planctomycetes*, *Proteobacteria* and *Verrucomicrobia* (Fig 6). Heatmap revealed that *Proteobacteria*, *Firmicutes*, *Bacteroidetes* and *Actinobacteria* were the predominant phyla in T, P, Y, Z and J (Fig 6). *Proteobacteria* and *Firmicutes* were found in all the samples especially in T1, T5 and J1, and *Bacteroidetes* and *Actinobacteria* were mainly found in T samples. *Crenarchaeota*, *Nitrospirae*, *Planctomycetes* and *Verrucomicrobia* were rarely detected except in T samples. The results suggested that *Proteobacteria* and *Firmicutes* adapt better to all environment, and *Bacteroidetes* and *Actinobacteria* adapted well in soil environment.

**Fig 7:**
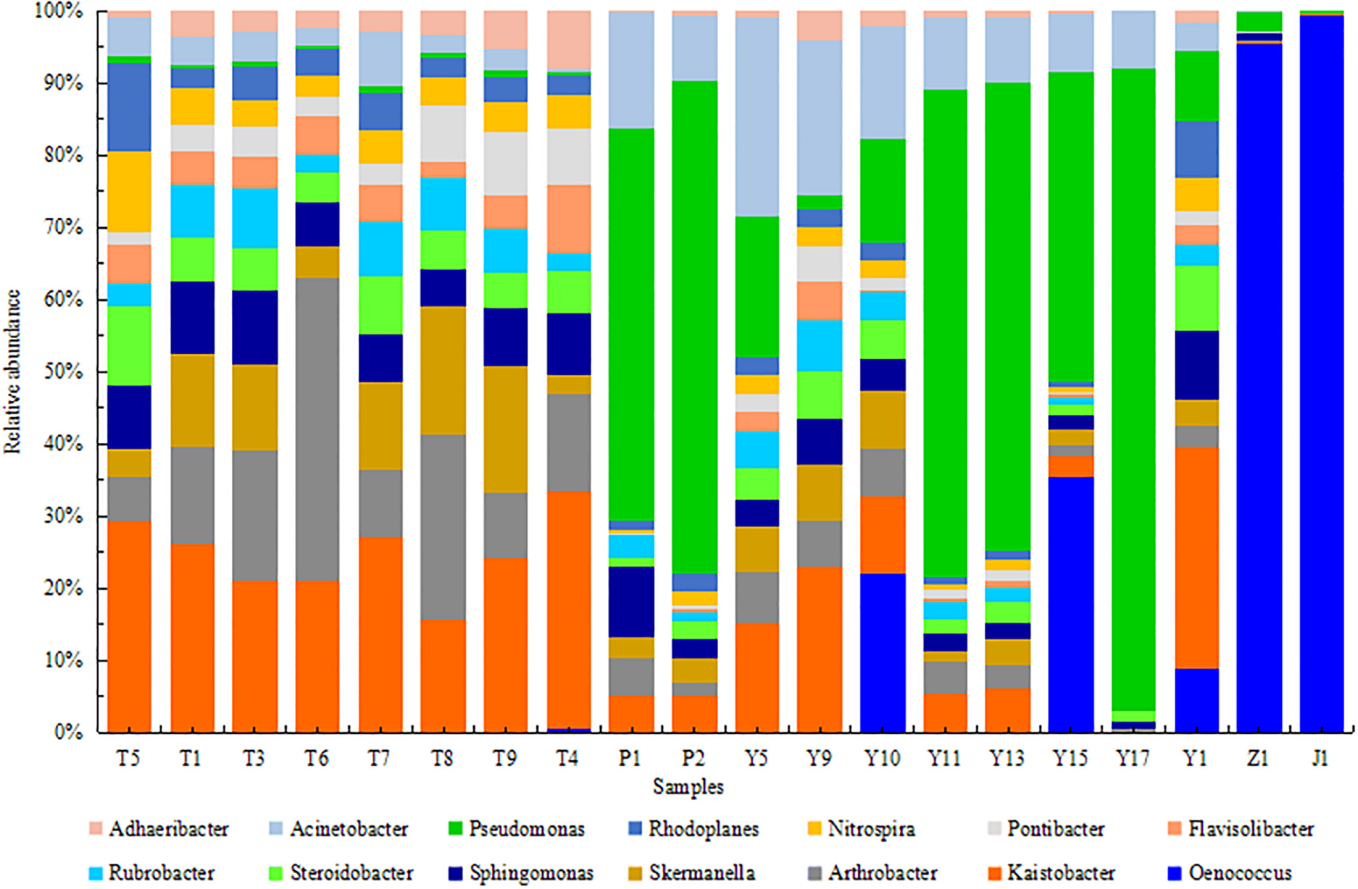
Relative abundance of bacteria at genus levels of T, P, Y, Z and J.

At the genus level, 317 bacterial genera were detected in T, P, Y, Z and J samples. The 14 bacterial genera with their relative abundance greater than 1% were selected (Fig 7). *Kaistobacter*, *Arthrobacter*, *Skermanella* and *Sphingomonas* were the predominant genera in T, *Pseudomonas*, *Acinetobacter* and *Kaistobacter* were the predominant genera in Y and P, and *Oenococcus* was the predominant genera in Z and J. Other major genera in samples include *Steroidobacter*, *Rubrobacter*, *Flavisolibacter*, *Pontibacter*, *Nitrospira*, *Rhodoplanes* and *Adhaeribacter*. These findings were consistent with previous reports [54–55]. The *Kaistobacter* abundance in October T samples was found increased compare with July sasmples, and *Arthrobacter* abundance was found declined. In contrast, no significant changes of *Skermanella* and *Sphingomonas* abundances in T samples were found. For Y and P samples collected in July and October, *Kaistobacter* abundance was not consistent, *Pseudomonas* and *Acinetobacter* in Y of A and B decreased in October. C sample was an exception that *Pseudomonas* and *Acinetobacter* have higher abundance in October samples, which might be due to interspecific completion that *Oenococcus* decreased from 35%(Y15) to 0%(Y17). *Oenococcus* was the dominant genus in Z and J samples, increased sharply to 95% in Z1 and 98% in J1. *Oenococcus* sp is a slow growing lactic acid bacterium. It is a necessary bacterium in winemaking process with the function of malolactic fermentation. It’s accumulation in wine is a natural process, and similar phenomenon was also observed in other reported studies [56]. *Lactobacillus plantarum* is another well studied bacterium with some strains been commercially used as malolactic fermentation (MLF) starter cultures. It is able to conduct MLF under high pH condition and in co-inoculation with yeasts. It has not been detected in this metagenomics analysis, possibly because of the special climate conditions in Xinjiang region is more suitable for *Oenococcus* sp.

It is discussed that the geographical location, natural climatic conditions [57], host plant phenology [58], physical and chemical properties of the soil [59–60], economic characteristics of the phyllosphere or soil [61], artificial vineyard management model, external pollution and other factors both can affected the T, Y and P microbial community composition to a certain extent, which in-turn determines the community species composition, quantity and distribution. The metagenomics analysis in this study indicates a strong correlation between grape and leave samples, differences of microbial communities in various regions were also discovered.

**Fig 8:**
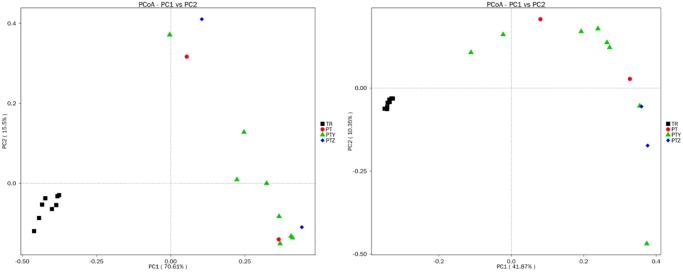
Principal coordinate analysis of bacteria microbial communities of samples.

**Fig 9:**
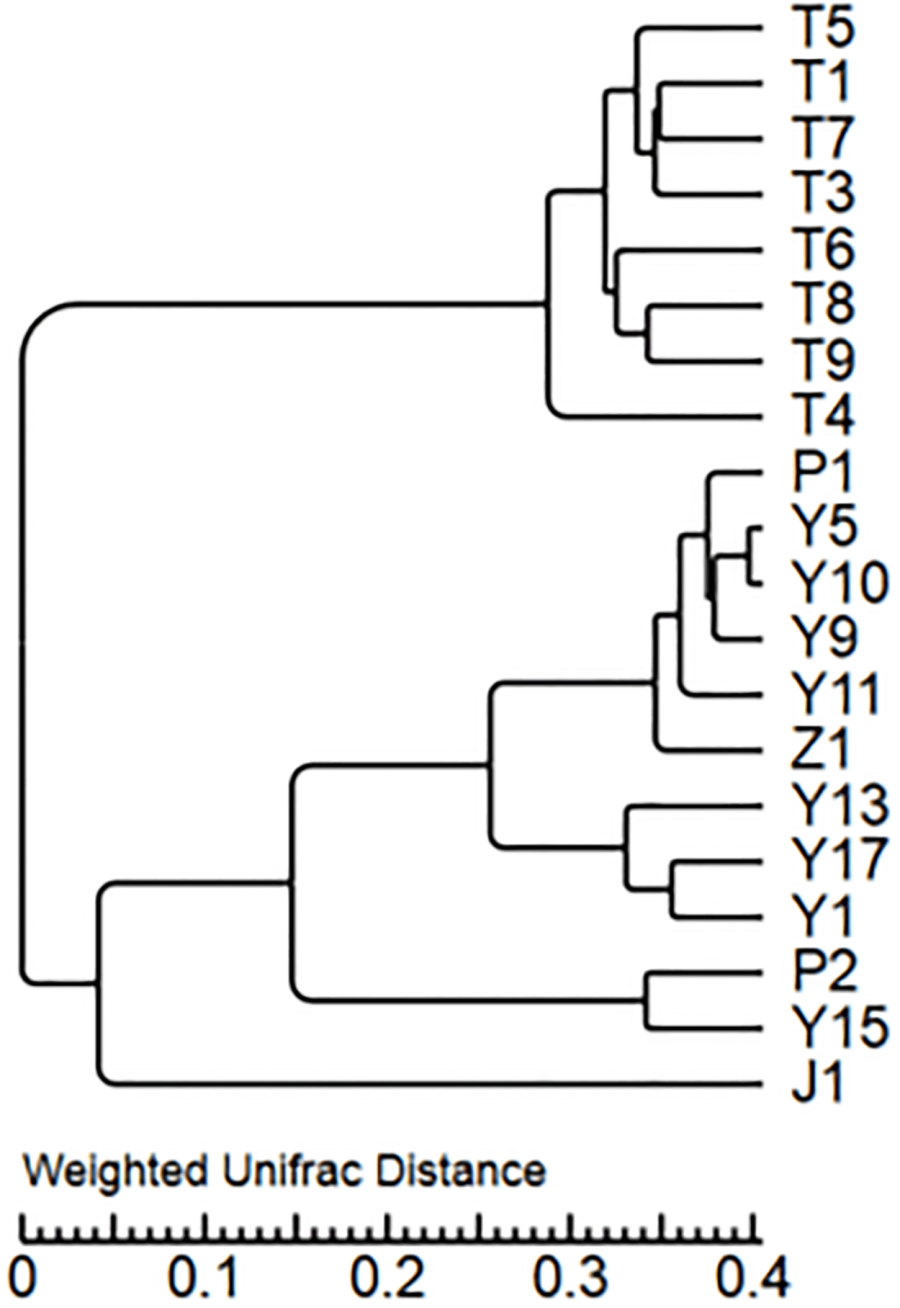
Cluster analysis of the bacterial microbiota.

PCoA of bacterial communities in T, Y, P, Z and J samples was performed using one weighted (PC1 variance = 70.61%, PC2 variance = 15.50%) and another weighted (PC1 variance = 41.87%, PC2 variance = 10.35%) (Fig 8). UPGMA cluster map (Fig 9) reflected the bacterial microbial community structure, indicate that the similarity of bacteria in all soils were higher except T4. P, Y, Z and J had some similarity in the bacterial microbial community, though differences were existed between vineyard management in the A, B, C region.

## Conclusions

The specific microbes in Xinjiang region served as autochthonous starter cultures, which play a major role in local winery production. In this study, microbial diversity of soil, grape, grape leaves, grape juice and wine were studied by high-throughput sequencing and identified the dominant genera and phyla. It is the first study to apply this technology on the winery research in Xinjiang region of China, aimed to understand the microbial diversity in Xinjiang region, and identify microorganisms that could improve the quality and flavor of wine product. The extensive revealing of microbial diversity in Xinjiang region is helpful in building wine microbial germplasm repository. In addition, this study identified various microbes which are beneficial for the wine production. Further identification and research on these microbes can help to figure out their indigenous beneficial nature, which will in-turn help the winery to improve quality and local characteristics of their wine product.

## Supporting information

S1 Fig The rarefaction curve of fungal. (TIF)

S2 Fig The rarefaction curve of bacterial. (TIF)

S1 Table The Flora diversity and abundance index of fungal. (XLS)

S2 Table The Flora diversity and abundance index of bacterial. (XLS)
