# Supplementary figures and images for: High-throughput sequencing of microbial community diversity in soil, grapes, leaves, grape juice and wine of grapevine from China
Source: PLoS One. 2018 Mar 22;13(3):e0193097. doi: 10.1371/journal.pone.0193097 (PMC5863948; doi:10.1371/journal.pone.0193097)

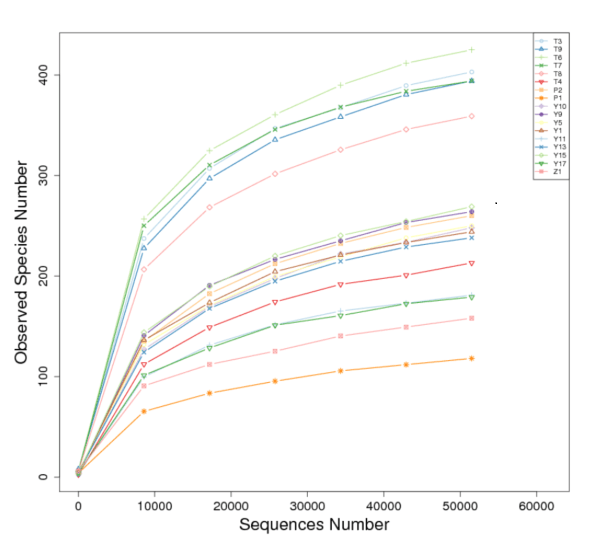

Supplement: S1 Fig — (TIF) [file pone.0193097.s001.tif]

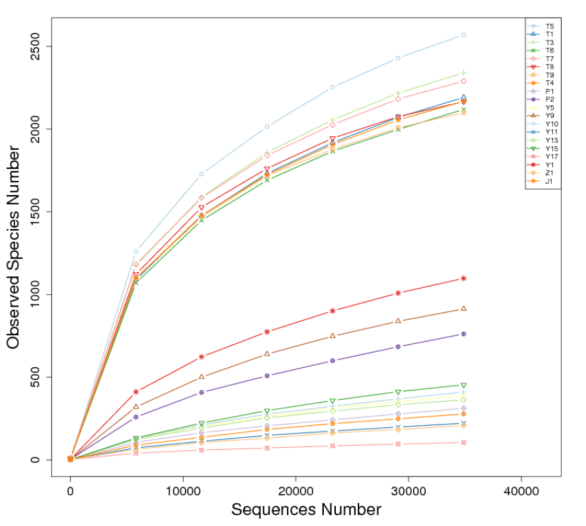

Supplement: S2 Fig — (TIF) [file pone.0193097.s002.tif]
